# Qualitative and Quantitative Analysis of Phytochemicals in Sayeok-Tang via UPLC-Q-Orbitrap-MS and UPLC-TQ-MS/MS

**DOI:** 10.3390/ph17091130

**Published:** 2024-08-27

**Authors:** Yu Jin Kim, Seol Jang, Youn-Hwan Hwang

**Affiliations:** KM Convergence Research Division, Korea Institute of Oriental Medicine, 1672 Yuseong-daero, Yuseong-gu, Daejeon 34054, Republic of Korea; swellseol@kiom.re.kr

**Keywords:** Sayeok-tang, UPLC-Q-Orbitrap-MS, UPLC-TQ-MS/MS, multiple reaction monitoring, quality control

## Abstract

Sayeok-tang (SYT) is a traditional herbal formula comprising three medicinal herbs: *Glycyrrhiza uralensis*, *Zingiber officinale*, and *Aconitum carmichaeli*. Several studies have employed liquid chromatography-mass spectrometry (LC-MS) to qualitatively analyze the components and metabolites of SYT in vitro and in vivo; however, studies on quantitative analysis of SYT, which is important for quality control, are absent or limited to only a few components. In this study, ultrahigh-performance liquid chromatography coupled with quadrupole (UPLC-Q)-Orbitrap-MS was used to screen the phytochemicals of SYT, revealing a total of 42 compounds. Among them, 24 compounds were simultaneously quantified within 20 min via UPLC-TQ-MS/MS in the multiple reaction monitoring mode. The developed analytical method was validated for its linearity (*r*^2^ ≥ 0.9992), precision (0.36–2.96%), accuracy (−6.52–4.64%), and recovery (94.39–119.07%) for all analytes, exhibiting acceptable results. The validated method was applied in the analysis of SYT extracts, and the 24 compounds were quantified in the range of 0.004–6.882 mg/g (CV ≤ 3.746%). Among them, liquiritin apioside (6.870–6.933 mg/g), glycyrrhizic acid (5.418–5.540 mg/g), and liquiritin (1.303–1.331 mg/g) from *G. uralensis* were identified as the relatively abundant compounds. The presented validated analytical method is highly promising for the comprehensive quality control of SYT, offering fast, highly sensitive, and reliable analysis.

## 1. Introduction

Sayeok-tang (SYT), known as Shigyaku-to in Japan and Sini-tang in China, is a traditional herbal formula of Shang Han Lun, comprising three medicinal herbs: *Glycyrrhiza uralensis*, *Zingiber officinale*, and *Aconitum carmichaeli* [1]. Previous studies have shown that SYT is effective in treating cardiovascular diseases, including the improvement of early ventricular remodeling and cardiac function in heart failure following myocardial infarction [2,3,4,5]. Clinical studies on the therapeutic effects of SYT on ischemia/reperfusion injury in patients with acute myocardial infarction and on angina pectoris in coronary artery disease have also been reported [6,7]. SYT has also been applied to improve lung injury caused by sepsis through various mechanisms. SYT ameliorates the symptoms and pathology associated with sepsis, such as pulmonary histopathological lesions in cecal ligation and puncture mice models by modulating gut microbiota [8] and improves sepsis-induced acute lung injury by regulating the ACE2-Ang (1–7)-Mas axis and inhibiting the mitogen-activated protein kinase signaling pathway [9]. Additionally, SYT has been shown to possess anti-inflammatory and antioxidant properties that attenuate acute lung injury induced by *E*. *coli* in mice [10]. A previous study predicted the association between SYT and ulcerative colitis (UC) through network pharmacology analysis and revealed the pharmacological effects of SYT on UC using rats with UC [11]. Although the various experimental and clinical efficacies of SYT are known, few studies report analytical methods for quality control of SYT.

The quality of herbal medicines contained in herbal formulas varies depending on various environmental factors; therefore, quality control is important to ensure their safety and efficacy. In recent years, ultrahigh-performance liquid chromatography coupled with high-resolution mass spectrometry (UPLC-HRMS) has become a powerful tool for chemical profiling of natural products [12]. In particular, UPLC coupled with quadrupole Orbitrap mass spectrometry (UPLC-Q-Orbitrap-MS) has been widely used to screen and identify phytochemicals in complex herbal samples owing to its excellent analytical sensitivity and specificity compared to other techniques, being ideal for identifying compounds by obtaining accurate molecular mass and multistage MS^n^ fragment ions of analytes [13,14,15]. Currently, UPLC coupled with triple quadrupole mass spectrometry (UPLC-TQ-MS/MS) has become a promising tool for simultaneous analysis of multiple target compounds in complex mixtures at low concentrations due to its high sensitivity and fast resolution [16,17]. The multiple reaction monitoring (MRM) mode of TQ-MS/MS is a rapid and highly sensitive analytical method that can selectively identify and quantify target compounds in complex mixtures by rapidly screening the transitions from specific precursor ions to product ions [17,18]. In addition, it is frequently applied to quantitative analysis in various research fields because it provides very low detection and quantitation limits without considering peak overlap interference [19,20,21]. Even though several studies have reported the qualitative analysis of the components and metabolites of SYT in vitro and in vivo using liquid chromatography-mass spectrometry (LC-MS), studies on quantitative analysis of SYT, which is important for quality control, are absent or limited to only a few components [22,23,24,25].

Therefore, in this study, a UPLC-Q-Orbitrap-MS method was applied to screen and characterize 42 phytochemicals of SYT by comparing retention times and MS information with reference standards. In addition, simultaneous quantification of 24 phytochemicals in SYT was performed using a validated UPLC-TQ-MS/MS method in the MRM mode, enabling rapid, sensitive, and high-throughput analysis. This study offers an efficient and reliable analytical method being a valuable tool for the comprehensive quality control of SYT.

## 2. Results and Discussion

### 2.1. Qualitative Analysis of SYT

**Figure 1:**
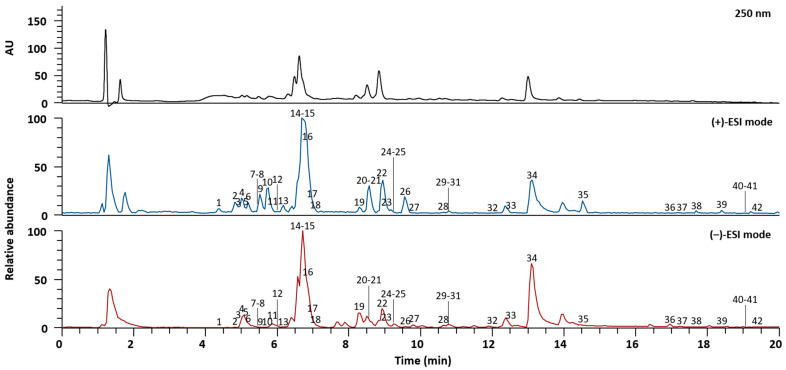
LC chromatogram and base peak chromatograms in the positive and negative ion modes of SYT extracts confirmed by UPLC-Q-Orbitrap-MS. Information on each compound corresponding to each number is presented in Table 1.

SYT extracts were analyzed via UPLC-Q-Orbitrap-MS to identify the phytochemicals attributed to the three herbal medicines: *G. uralensis*, *Z. officinale*, and *A. carmichaeli* [26]. The different compounds were separated within 20 min using an Acquity BEH C_18_ column (100 × 2.1 mm, 1.7 µm, Waters, Milford, MA, USA) with gradient elution of 0.1% (*v*/*v*) aqueous formic acid and acetonitrile. Both the positive and negative ESI modes were used to acquire MS spectra. A total of 42 compounds, including vicenin-2, schaftoside, daidzin, neoliquiritin, liquiritin apioside, liquiritin, ferulic acid, genistin, isoliquiritin apioside, isoliquiritin, ononin, licochalcone B, liquiritigenin, licochalcone A, genistein, naringenin, echinatin, isoliquiritigenin, formononetin, glycyrrhizic acid, glabridin, and glycyrrhetinic acid from *G. uralensis* [27], 6-gingerol, 8-gingerol, 6-shogaol, diacetoxy-6-gingerdiol, 10-gingerol, and 8-shogaol from *Z. officinale* [28], and karacolidine, mesaconine, senbusine A, karacoline, aconine, napellonine, hypaconine, fuziline, bullatine B, talatisamine, benzoylmesaconine, benzoylaconine, benzoylhypacoitine, and hypaconitine from *A. carmichaeli* [29,30,31], were identified by comparing their retention times, precursor ions, and MS/MS fragments to those of reference standards. The characteristics of all the identified compounds in SYT based on MS data are summarized in Table 1. Alkaloids from *A. carmichaeli* and phenols from *Z. officinale* were clearly detected in the positive ion mode, whereas the compounds from *G. uralensis* were ionized in similar proportions in the positive and negative ion modes. The LC chromatogram at 250 nm and base peak chromatograms in the positive and negative ion modes of SYT extracts are presented in Figure 1.

### 2.2. Quantitative Analysis

**Figure 2:**
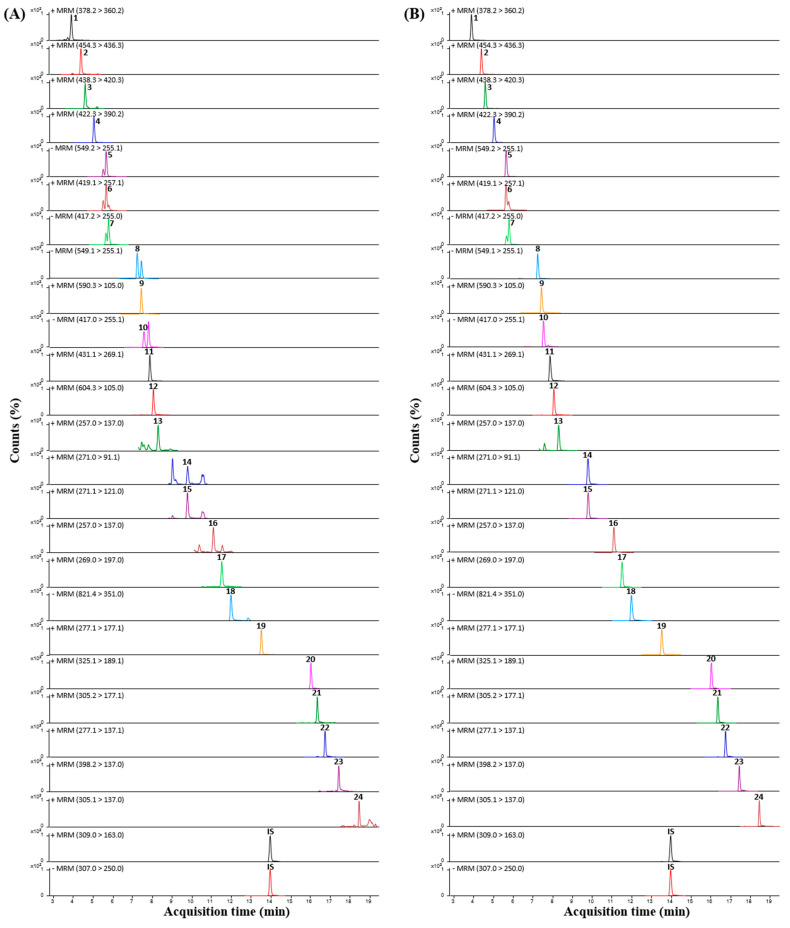
Multiple reaction monitoring (MRM) chromatograms of the 24 compounds in the (**A**) SYT extracts and (**B**) standard mixture.

To quantify the 24 phytochemicals identified in the SYT extracts, UPLC-TQ-MS/MS analysis was performed in dynamic MRM mode optimized for each analyte, and all analytes were detected within 20 min under 0.1% (*v*/*v*) aqueous formic acid-acetonitrile gradient conditions. The MRM mode of TQ-MS/MS is an ideal method for selectively identifying and quantifying compounds in complex mixtures by rapidly screening for transitions from specific precursor ions to product ions [18]. The optimized MRM parameters for each of the 24 compounds and internal standards (IS), including ionization mode, MRM transitions, and collision energy, are summarized in Table 2. The retention times, precursor ions, and product ions of each analyte were compared to those of reference standards. Most analytes were detected in the positive ion mode, while five analytes, liquiritin apioside, liquiritin, isoliquiritin apioside, isoliquiritin, and glycyrrhizic acid, were more suitably ionized in the negative ion mode. The MRM chromatograms of the analytes in the positive or negative ion modes are shown in Figure 2.

The MS fragmentation patterns from the precursor ions to the dominant product ions were confirmed through UPLC-TQ-MS/MS analysis in the dynamic MRM mode. The six *Aconitum* alkaloids, karacoline, fuziline, bullatine B, talatisamine, benzoylmesaconine, and benzoylaconine, exhibited protonated molecular ions [M + H]^+^ at *m*/*z* 378.2, 454.3, 438.3, 422.3, 590.3, and 604.3, respectively. Karacoline, fuziline, and bullatine B lost a water molecule (18 Da) from their precursor ions to form [M + H − H_2_O]^+^ ions at *m*/*z* 360.2, 436.3, and 420.3, respectively [30,31,32]. Talatisamine generated a fragment ion [M + H − CH_3_OH]^+^ at *m*/*z* 390.2 by losing a methanol molecule (32 Da) from the precursor ion. Benzoylmesaconine and benzoylaconine generated a product ion at *m*/*z* 105.0, corresponding to the benzoyl group [33]. Among the 13 constituents of *G. uralensis*, five compounds, liquiritin apioside, liquiritin, isoliquiritin apioside, isoliquiritin, and glycyrrhizic acid, exhibited [M − H]^−^ ions at *m*/*z* 549.2, 417.2, 549.1, 417.0, and 821.4, respectively. Liquiritin and isoliquiritin generated [M − H − Glc]^−^ ions at *m*/*z* 255.0 and 255.1, respectively, which resulted from the loss of glucose (162 Da). In the case of liquiritin apioside and isoliquiritin apioside, a fragment ion [M − H − Api − Glc]^−^ was produced at *m*/*z* 255.1 by losing an apiosyl glucoside from the precursor ion. Glycyrrhizic acid produced a fragment ion [2GluA − H]^−^ at *m*/*z* 351.0, indicating the loss of two glucuronic acids [34]. In the positive ion mode, protonated molecular ions [M + H]^+^ of the remaining eight compounds from *G. uralensis* were observed. For neoliquiritin and ononin, the precursor ions at *m*/*z* 419.1 and 431.1 eliminated a glucose molecule (162 Da) to generate fragment ions [M + H − Glc]^+^ at *m*/*z* 257.1 and 269.1, respectively. Liquiritigenin and isoliquiritigenin exhibited [M + H]^+^ ions at *m*/*z* 257.0 and had the same fragment ions [M + H − C_8_H_8_O]^+^ at *m*/*z* 137.0 [35,36]. The precursor ion [M + H]^+^ of formononetin observed at *m*/*z* 269.0 subsequently underwent several fragmentations, including loss of CH_4_ (16 Da) and 2CO (56 Da), to generate a specific fragment ion [M + H − C_3_H_4_O_2_]^+^ at *m*/*z* 197.0 [37]. The fragment ions of echinatin at *m*/*z* 121.0 and genistein at *m*/*z* 91.1 were generated from the precursor ions [M + H]^+^ at *m*/*z* 271.1 and 271.0, respectively [38,39]. Regarding glabridin, a characteristic fragment ion [M + H − C_8_H_8_O_2_]^+^ was identified at *m*/*z* 189, generated by a Retro-Diels-Alder reaction from the precursor ion at *m*/*z* 325.1 [M + H]^+^ [40,41]. The precursor ions of 6-gingerol and 8-gingerol in the form [M + H − H_2_O]^+^ were identified at *m*/*z* 277.1 and 305.2, respectively, while the [M + H − H_2_O − C_6_H_12_O]^+^ and [M + H − H_2_O − C_8_H_16_O]^+^ fragment ions were generated at *m*/*z* 177.1, respectively, by the loss of the neutral alkyl moiety and rearrangement [42]. Diacetoxy-6-gingerdiol exhibited an *m*/*z* 398.2 [M + NH_4_]^+^ and fragment ion at *m*/*z* 137.0. Regarding 6-shogaol and 8-shogaol, the precursor ions [M + H]^+^ were observed at *m*/*z* 277.1 and 305.1, respectively, and the fragment ions [M + H − C_9_H_16_O]^+^ and [M + H − C_11_H_20_O]^+^ were produced at *m*/*z* 137.1 and 137.0, respectively [28].

### 2.3. Method Validation for Quantitative Analysis

The linearity, limits of detection (LOD) and quantification (LOQ), precision, accuracy, and recovery were evaluated to validate the developed analytical method. The calibration curves for each analyte were linear over a wide concentration range and observed appropriate results without weighting compared to using weighting factors such as 1/*x*, 1/*x*^2^, 1/*y*, or 1/*y*^2^. The correlation coefficients are within the acceptable limits (*r*^2^ ≥ 0.9992). The LODs and LOQs of the 24 analytes ranged from 0.007–5.165 ng/mL and 0.020–15.651 ng/mL, respectively. The linear ranges, regression equations, correlation coefficient values, LODs, and LOQs of the 24 compounds are listed in Table 3. Precision was expressed as the coefficient of variation (CV) (%) of the observed concentration values for six replicates of the reference standards at three concentration levels (low, medium, and high). The intra- and inter-day precisions of the 24 compounds were less than 2.54% and 2.96%, respectively, and the accuracies, expressed as the relative error (RE) (%), ranged from −6.52 to 4.37% and −5.41 to 4.64%, respectively (Table 4). Recovery tests were performed by adding the standard solutions of the 24 compounds at three different concentrations (low, medium, and high) to the original sample of known concentration (Table 5). The recovery (%) of all analytes ranged from 94.39 to 119.07% (CV ≤ 4.75%). These verified results demonstrate that the established UPLC-TQ-MS/MS method exhibits acceptable linearity, sensitivity, precision, accuracy, and recovery and is suitable for the quantitative analysis of 24 phytochemicals in SYT.

### 2.4. Quantification of 24 Phytochemicals in SYT

The validated UPLC-TQ-MS/MS method in MRM mode was subsequently applied to the quantitative analysis of 24 phytochemicals in three batches of SYT samples. The contents of the 24 compounds were measured in the range of 0.004 to 6.882 mg/g (CV ≤ 3.746%) based on the calibration curve, and the average contents of each batch for all analytes are presented in Table 6. Among these compounds, liquiritin apioside (6.870–6.933 mg/g), glycyrrhizic acid (5.418–5.540 mg/g), and liquiritin (1.303–1.331 mg/g) from *G. uralensis* were relatively abundant in all three batches of SYT samples.

Several researchers have reported in previous studies that the contents of the components in the three herbal medicines of SYT vary depending on seasonal and geographical factors [43,44,45,46,47]. The content and composition of SYT ingredients may be influenced by environmental changes, geographical location, soil conditions, and harvest time. These influence factors can affect the overall quality and efficacy of herbal medicines [48,49]. Although we have developed and validated fast and sensitive UPLC-MS-based methods for the quality control in SYT, the evaluation of its phytochemical diversity and complexity considering various influence factors were not included in this study. In this regard, further studies are required to investigate various seasonality or to compare with other blends coming from geographical locations with different characteristics. Therefore, our precise and sensitive analytical methods can provide sufficiently valuable and helpful information for investigating various subsequent studies of SYT quality control.

## 3. Materials and Methods

### 3.1. Materials and Reagents

The three herbal medicines included in SYT, *Glycyrrhiza uralensis*, *Zingiber officinale*, and *Aconitum carmichaeli*, were purchased from the herbal medicine market Kwangmyungdang Pharmaceutical (Ulsan, Republic of Korea), and the voucher specimens were deposited at the KM Convergence Research Division of the Korea Institute of Oriental Medicine (Daejeon, Republic of Korea). The 42 reference standards (purity ≥ 95%) used in the qualitative analysis of SYT were purchased from TargetMol (Boston, MA, USA). The 24 reference standards (purity ≥ 98%), karacoline, fuziline, bullatine B, talatisamine, liquiritin apioside, neoliquiritin, liquiritin, isoliquiritin apioside, benzoylmesaconine, isoliquiritin, ononin, benzoylaconine, liquiritigenin, echinatin, genistein, isoliquiritigenin, formononetin, glycyrrhizic acid, 6-gingerol, glabridin, 8-gingerol, 6-shogaol, diacetoxy-6-gingerdiol, and 8-shogaol were purchased from ChemFaces Biochemical (Wuhan, China) and used for quantitative analysis. Warfarin was used as IS and was obtained from Sigma-Aldrich (St. Louis, MO, USA). Methanol, water, acetonitrile, and formic acid (LC-MS grade) were purchased from Thermo Fisher Scientific (Waltham, MA, USA).

### 3.2. Preparation of Standard Solutions

The 24 reference standards and warfarin (IS) were each prepared at a concentration of 1.0 mg/mL in methanol. These stock solutions were then further diluted with methanol to obtain a series of standard solutions for the calibration curves and method validation. The concentration of IS was consistently fixed at 5.0 ng/mL in all standard solutions.

### 3.3. Extraction of SYT

SYT (228 g), containing a mixture of the three herbal medicines *Glycyrrhiza uralensis*, *Zingiber officinale*, and *Aconitum carmichaeli* in a ratio of 1:1.5:0.75, was extracted via refluxing with distilled water at 100 °C for 3 h. The extract solution was filtered, concentrated using a rotary evaporator system under vacuum, and freeze-dried to obtain a powdered extract (57.72 g, 25.32%). The powdered SYT extract was dissolved in methanol at a concentration of 50 μg/mL, filtered through a syringe filter (0.2 μm pore size), and used as a sample solution for analysis.

### 3.4. UPLC-Q-Orbitrap-MS Conditions

Qualitative analysis of SYT was performed using a Dionex UltiMate 3000 system connected to a Thermo Q-Exactive mass spectrometer (Thermo Fisher Scientific, Waltham, MA, USA) equipped with an electrospray ionization (ESI) source according to the previously reported methods [50]. The phytochemicals in SYT were identified by gradient elution of 0.1% (*v*/*v*) aqueous formic acid and acetonitrile on an Acquity BEH C_18_ column (100 × 2.1 mm, 1.7 µm, Waters, Milford, MA, USA) maintained at 40 °C. MS analysis was conducted with an ESI source in both the positive and negative modes and MS spectra were acquired at a normalized collision energy of 25 eV in full MS-ddMS^2^ mode over a scan range of 100–1500 *m*/*z*. The source parameters were set as follows: ion spray voltage, 3.8 kV; capillary temperature, 320 °C; sheath gas pressure, 40 arbitrary units (au); auxiliary gas pressure, 10 au; Slens RF level, 60; and resolution, 70,000 (full MS) and 17,500 (ddMS^2^). All data were processed using Thermo Xcalibur v.3.0 and Tracefinder v.3.2 (Thermo Fisher Scientific, Bremen, Germany).

### 3.5. UPLC-TQ-MS/MS Conditions

Quantitative analysis of the 24 compounds in SYT was performed with an Agilent 1290 Infinity II UPLC system equipped with a 6495C triple quadrupole mass spectrometer (Agilent Technologies, Santa Clara, CA, USA) with a jet-stream ESI source. The 24 compounds were separated on an Acquity BEH C_18_ column (100 × 2.1 mm, 1.7 µm, Waters, Milford, MA, USA) maintained at 40 °C by gradient elution of 0.1% (*v*/*v*) aqueous formic acid (A) and acetonitrile (B) using the following method: 3% B for 0–1 min, 3–15% B for 1–2 min, 15–50% B for 2–13 min, 50–100% B for 13–20 min, and 100% B for 20–23 min at a flow rate of 0.25 mL/min. The mass spectrometer was operated in the dynamic MRM mode, and the MRM data were collected in the positive or negative ion mode depending on the optimal ionization conditions for each compound. The ESI source conditions involved a drying gas temperature of 130 °C, drying gas flow of 11 L/min, nebulizer pressure of 25 psi, sheath gas temperature of 400 °C, sheath gas flow of 12 L/min, capillary voltage of 3500 V (positive) and 3000 V (negative), and nozzle voltage of 500 V (positive) and 1500 V (negative). Agilent MassHunter Workstation v.10.1 software (Agilent Technologies, Santa Clara, CA, USA) was used for all data acquisition and processing.

### 3.6. Validation of the UPLC-TQ-MS/MS Method

Calibration curves of the 24 reference standards were established from the peak areas of standard solutions at nine different concentration levels, and the linear relationships between the peak area (*y*) and corresponding concentration (*x*, ng/mL) of each standard were expressed via the regression equation (*y* = a*x* + b). Standard solutions were measured five times repeatedly to obtain the calibration curves. The LOD and LOQ for the 24 compounds were calculated using the slope of the calibration curve and the standard deviation (SD) of the intercept as follows: LOD = 3.3 × (SD of the response/slope of the calibration curve) and LOQ = 10 × (SD of the response/slope of the calibration curve). To assess precision, three standard solutions containing low, medium, and high concentrations of each standard were analyzed repeatedly (*n* = 6) in one day and three consecutive days to measure the intra- and inter-day variation. Precision was expressed as CV (%) of the measured concentration values and calculated using the following formula: CV (%) = (SD/Mean) × 100. Accuracy was represented by RE (%) and calculated as follows: RE (%) = (observed concentration − expected concentration)/expected concentration × 100. Recovery tests were performed by spiking standard solutions of three different concentrations (low, medium, and high) into samples of known concentration. The recovery (%) was calculated according to the following equation: recovery (%) = (found concentration − original concentration)/spiked concentration × 100.

## 4. Conclusions

The phytochemicals of SYT were studied via UPLC-Q-Orbitrap-MS analyses, and a total of 42 compounds were identified in the positive and negative ESI modes. The qualitative analysis results, including retention time and MS data, were compared with those of reference standards. Within 20 min, 24 compounds were simultaneously quantified in the MRM mode using the optimized UPLC-TQ-MS/MS method. The method was validated for its linearity, precision, accuracy, and recovery, exhibiting acceptable results and confirming that the established analytical method is suitable for quantifying the components of SYT. Our study offers a valuable tool for the comprehensive quality control of SYT.

## Figures and Tables

**Table 1:** Phytochemicals identified in SYT via UPLC-Q-Orbitrap-MS analysis.

No.	RT (min)	Precursor Ion (*m/z*)			Error (ppm)	Formula	MS/MS Fragments (*m*/*z*)	Identifications
		Calculated	Estimated	Adduct				
**1**	4.40	394.2595	394.2588	M + H	1.8300	C_22_H_35_NO_5_	394.2594, 376.2486, 238.1674	Karacolidine
**2**	4.82	486.2708	486.2698	M + H	2.2282	C_24_H_39_NO_9_	468.2529, 454.2444, 436.2336	Mesaconine
**3**	4.91	424.2701	424.2694	M + H	1.6264	C_23_H_37_NO_6_	424.2702, 406.2595, 388.2481	Senbusine A
**4**	5.01	378.2646	378.2639	M + H	1.8437	C_22_H_35_NO_4_	360.2533, 243.3279, 127.9954	Karacoline
**5**	5.15	500.2866	500.2854	M + H	2.4208	C_25_H_41_NO_9_	420.2416, 402.2276, 276.1242	Aconine
**6**	5.19	358.2385	358.2377	M + H	2.2174	C_22_H_31_NO_3_	358.2383, 340.2278, 191.1758	Napellonine
**7**	5.45	593.1526	593.1512	M − H	2.4596	C_27_H_30_O_15_	473.1106, 383.0779, 353.0671	Vicenin-2
**8**	5.47	470.2759	470.2748	M + H	2.2091	C_24_H_39_NO_8_	470.2759, 438.2494, 310.1442	Hypaconine
**9**	5.52	454.2809	454.2799	M + H	2.2339	C_24_H_39_NO_7_	454.2809, 436.2684	Fuziline
**10**	5.76	438.2860	438.2850	M + H	2.3529	C_24_H_39_NO_6_	438.2859, 420.2757, 388.2509	Bullatine B
**11**	5.87	563.1420	563.1406	M − H	2.3756	C_26_H_28_O_14_	503.1196, 443.0996, 353.0671	Schaftoside
**12**	5.99	417.1189	417.1180	M + H	2.0521	C_21_H_20_O_9_	416.2454, 255.0655, 137.0236	Daidzin
**13**	6.18	422.2907	422.2901	M + H	1.4456	C_24_H_39_NO_5_	422.2909, 390.2650, 258.0841	Talatisamine
**14**	6.69	419.1343	419.1337	M + H	1.5457	C_21_H_22_O_9_	257.0812, 147.0446, 137.0237	Neoliquiritin
**15**	6.72	549.1624	549.1614	M − H	1.8092	C_26_H_30_O_13_	255.0665, 135.0076, 119.0491	Liquiritin apioside
**16**	6.85	417.1198	417.1191	M − H	1.5367	C_21_H_22_O_9_	255.0665, 135.0076, 119.0490	Liquiritin
**17**	6.99	193.0502	193.0506	M − H	−2.2201	C_10_H_10_O_4_	178.0264, 149.0598, 134.0362	Ferulic acid
**18**	7.07	477.1046	477.1038	M + HCO_2_	1.6019	C_21_H_20_O_10_	431.0991, 269.0459, 255.0665	Genistin
**19**	8.31	549.1626	549.1614	M − H	2.2537	C_26_H_30_O_13_	255.0664, 151.0390, 135.0075	Isoliquiritin apioside
**20**	8.57	590.2971	590.2960	M + H	1.8433	C_31_H_43_NO_10_	558.2698, 540.2626, 105.0343	Benzoylmesaconine
**21**	8.60	417.1199	417.1180	M − H	4.4697	C_21_H_22_O_9_	297.0777, 255.0664, 135.0076	Isoliquiritin
**22**	8.92	431.1345	431.1337	M + H	1.9274	C_22_H_22_O_9_	269.0812	Ononin
**23**	9.01	285.0774	285.0768	M − H	1.7962	C_16_H_14_O_5_	285.0771, 270.0537, 150.0312	Licochalcone B
**24**	9.18	604.3130	604.3116	M + H	2.2642	C_32_H_45_NO_10_	554.2754, 501.9368, 269.0811	Benzoylaconine
**25**	9.25	257.0814	257.0808	M + H	2.2596	C_15_H_12_O_4_	239.0709, 147.0445, 137.0237	Liquiritigenin
**26**	9.56	574.3024	574.3011	M + H	2.3841	C_31_H_43_NO_9_	574.3021, 542.2756, 147.0821	Benzoylhypacoitine
**27**	9.79	616.3131	616.3116	M + H	2.4182	C_33_H_45_NO_10_	488.3347, 411.4201, 313.6526	Hypaconitine
**28**	10.65	339.1600	339.1591	M + H	2.6039	C_21_H_22_O_4_	215.1073, 163.0758, 137.0601	Licochalcone A
**29**	10.72	271.0606	271.0601	M + H	1.8738	C_15_H_10_O_5_	229.0865, 153.0186, 121.0290	Genistein
**30**	10.76	271.0618	271.0612	M − H	2.2065	C_15_H_12_O_5_	177.0184, 151.0026, 119.0489	Naringenin
**31**	10.79	271.0971	271.0965	M + H	2.1799	C_16_H_14_O_4_	229.0865, 153.0186, 121.0290	Echinatin
**32**	12.00	257.0813	257.0808	M + H	1.6661	C_15_H_12_O_5_	239.0707, 147.0444, 137.0237	Isoliquiritigenin
**33**	12.50	269.0813	269.0808	M + H	1.7052	C_16_H_12_O_4_	254.0571, 237.0545, 137.0595	Formononetin
**34**	13.09	821.3982	821.3965	M − H	2.0494	C_42_H_62_O_16_	776.1565, 351.0583, 193.0348	Glycyrrhizic acid
**35**	14.52	277.1804	277.1798	M − H_2_O + H	1.9400	C_17_H_26_O_4_	177.0914, 145.0652, 137.0601	6-Gingerol
**36**	16.95	325.1440	325.1434	M + H	1.7606	C_20_H_20_O_4_	189.0914, 149.0601, 123.0446	Glabridin
**37**	17.31	305.2118	305.2111	M − H_2_O + H	2.1976	C_19_H_30_O_4_	177.0914, 145.0652, 137.0601	8-Gingerol
**38**	17.69	277.1804	277.1798	M + H	1.9442	C_17_H_24_O_3_	137.0601	6-Shogaol
**39**	18.40	398.2547	398.2537	M + NH_4_	2.3977	C_21_H_32_O_6_	261.1853, 163.0757, 137.0601	Diacetoxy-6-gingerdiol
**40**	19.06	471.3479	471.3469	M + H	2.0789	C_30_H_46_O_4_	267.0661, 235.1690, 189.1646	Glycyrrhetinic acid
**41**	19.06	373.2356	373.2349	M + Na	1.8654	C_21_H_34_O_4_	218.1184, 159.0420, 129.0550	10-Gingerol
**42**	19.39	305.2118	305.2111	M + H	2.2017	C_19_H_28_O_3_	137.0600	8-Shogaol

**Table 2:** Optimized MRM parameters for the 24 compounds in SYT extracts.

No.	Compound	RT (min)	Molecular Weight	Polarity	MRM Transition (*m*/*z*)	Collision Energy (V)
**1**	Karacoline	3.91	377.5	Positive	378.2 → 360.2	30
**2**	Fuziline	4.39	453.6	Positive	454.3 → 436.3	34
**3**	Bullatine B	4.60	437.6	Positive	438.3 → 420.3	30
**4**	Talatisamine	5.04	421.6	Positive	422.3 → 390.2	30
**5**	Liquiritin apioside	5.65	550.5	Negative	549.2 → 255.1	34
**6**	Neoliquiritin	5.65	418.4	Positive	419.1 → 257.1	10
**7**	Liquiritin	5.79	418.4	Negative	417.2 → 255.0	18
**8**	Isoliquiritin apioside	7.24	550.5	Negative	549.1 → 255.1	30
**9**	Benzoylmesaconine	7.44	589.7	Positive	590.3 → 105.0	40
**10**	Isoliquiritin	7.58	418.4	Negative	417.0 → 255.1	18
**11**	Ononin	7.87	430.4	Positive	431.1 → 269.1	18
**12**	Benzoylaconine	8.06	603.7	Positive	604.3 → 105.0	40
**13**	Liquiritigenin	8.30	256.3	Positive	257.0 → 137.0	26
**14**	Echinatin	9.78	270.3	Positive	271.1 → 121.0	26
**15**	Genistein	9.80	270.2	Positive	271.0 → 91.1	40
**16**	Isoliquiritigenin	11.09	256.3	Positive	257.0 → 137.0	22
**17**	Formononetin	11.52	268.3	Positive	269.0 → 197.0	40
**18**	Glycyrrhizic acid	11.98	822.9	Negative	821.4 → 351.0	40
**19**	6-Gingerol	13.52	294.4	Positive	277.1 → 177.1	10
**20**	Glabridin	16.01	324.4	Positive	325.1 → 189.1	14
**21**	8-Gingerol	16.35	322.4	Positive	305.2 → 177.1	10
**22**	6-Shogaol	16.74	276.4	Positive	277.1 → 137.1	10
**23**	Diacetoxy-6-gingerdiol	17.44	380.5	Positive	398.2 → 137.0	30
**24**	8-Shogaol	18.45	304.4	Positive	305.1 → 137.0	14
**IS**	Warfarin	13.98	307.1	Positive	309.0 → 163.0	14
**IS**	Warfarin	13.98	307.1	Negative	307.0 → 250.0	22

**Table 3:** Regression equations, linear ranges, correlation coefficients, LODs, and LOQs of the 24 compounds present in SYT.

No.	Compound	Linear Range (ng/mL)	Regression Equation (*y* = a*x* + b) ^a^	Correlation Coefficient (*r*^2^)	LOD ^b^ (ng/mL)	LOQ ^c^ (ng/mL)
**1**	Karacoline	0.024–6.25	*y* = 0.246822*x* − 0.001428	0.9995	0.024	0.071
**2**	Fuziline	0.024–6.25	*y* = 0.365585*x* − 0.002038	0.9994	0.068	0.207
**3**	Bullatine B	0.049–12.5	*y* = 0.175573*x* − 0.002382	0.9993	0.051	0.154
**4**	Talatisamine	0.024–6.25	*y* = 0.317425*x* − 0.001548	0.9997	0.017	0.051
**5**	Liquiritin apioside	3.125–800	*y* = 0.149587*x* − 0.041386	0.9999	2.753	8.341
**6**	Neoliquiritin	0.781–200	*y* = 0.038745*x* − 0.005777	0.9993	1.337	4.050
**7**	Liquiritin	1.563–400	*y* = 0.373646*x* − 0.036446	0.9998	1.670	5.059
**8**	Isoliquiritin apioside	0.195–50	*y* = 0.168865*x* − 0.002181	0.9997	0.169	0.513
**9**	Benzoylmesaconine	0.098–25	*y* = 0.075469*x* − 0.001463	0.9995	0.150	0.456
**10**	Isoliquiritin	0.195–50	*y* = 0.212640*x* − 0.002329	0.9995	0.198	0.601
**11**	Ononin	0.781–200	*y* = 0.294159*x* − 0.009772	0.9995	0.915	2.772
**12**	Benzoylaconine	0.024–6.25	*y* = 0.018718*x* − 0.000024	0.9995	0.023	0.070
**13**	Liquiritigenin	0.049–12.5	*y* = 0.120708*x* − 0.000840	0.9995	0.080	0.242
**14**	Echinatin	0.024–6.25	*y* = 0.645024*x* − 0.001478	0.9995	0.037	0.113
**15**	Genistein	0.024–6.25	*y* = 0.062600*x* − 0.000374	0.9994	0.024	0.072
**16**	Isoliquiritigenin	0.012–3.125	*y* = 0.125080*x* + 0.000054	0.9997	0.007	0.020
**17**	Formononetin	0.012–3.125	*y* = 0.613307*x* − 0.001759	0.9995	0.028	0.084
**18**	Glycyrrhizic acid	3.125–800	*y* = 0.061782*x* − 0.013360	0.9997	5.165	15.651
**19**	6-Gingerol	0.781–200	*y* = 0.099518*x* − 0.015199	0.9992	1.033	3.131
**20**	Glabridin	0.049–12.5	*y* = 0.192746*x* − 0.000652	0.9997	0.059	0.180
**21**	8-Gingerol	0.049–12.5	*y* = 0.071919*x* + 0.000108	0.9995	0.047	0.144
**22**	6-Shogaol	0.098–25	*y* = 0.275032*x* − 0.004930	0.9994	0.138	0.417
**23**	Diacetoxy-6-gingerdiol	0.049–12.5	*y* = 0.389618*x* − 0.002943	0.9997	0.032	0.098
**24**	8-Shogaol	0.024–6.25	*y* = 0.106655*x* − 0.000004	0.9997	0.027	0.080

^a^ *y* = a*x* + b, *y* indicates peak area and *x* indicates concentration (ng/mL). ^b^ LOD: 3.3 × (standard deviation (SD) of the response/slope of the calibration curve). ^c^ LOQ: 10 × (SD of the response/slope of the calibration curve).

**Table 4:** Precision and accuracy data for the 24 compounds in SYT.

No.	Compound	Conc. (ng/mL)	Intra-Day (*n* = 6)			Inter-Day (*n* = 6)		
			Observed Conc. (ng/mL)	CV ^a^ (%)	RE ^b^ (%)	Observed Conc. (ng/mL)	CV (%)	RE (%)
**1**	Karacoline	0.52	0.53	0.94	1.07	0.53	1.23	1.17
		2.08	2.10	0.77	0.76	2.09	1.08	0.49
		4.17	4.14	1.07	−0.71	4.04	2.29	−3.00
**2**	Fuziline	0.52	0.52	0.87	−0.20	0.53	1.34	1.27
		2.08	2.09	0.67	0.36	2.14	2.42	2.80
		4.17	4.07	1.39	−2.40	4.06	2.10	−2.55
**3**	Bullatine B	1.04	1.05	0.83	0.35	1.05	0.84	0.87
		4.17	4.20	0.48	0.74	4.29	2.50	2.92
		8.33	8.14	1.02	−2.29	8.02	2.06	−3.77
**4**	Talatisamine	0.52	0.52	1.11	−0.04	0.52	0.95	0.43
		2.08	2.09	0.39	0.30	2.13	1.95	2.30
		4.17	4.02	0.60	−3.41	4.03	0.98	−3.37
**5**	Liquiritin apioside	66.67	65.93	0.47	−1.10	66.03	0.91	−0.95
		266.67	262.40	0.71	−1.60	261.14	0.73	−2.07
		533.33	538.76	0.76	1.02	537.34	0.94	0.75
**6**	Neoliquiritin	16.67	16.73	0.56	0.35	16.72	0.73	0.32
		66.67	68.17	1.00	2.26	69.76	2.27	4.64
		133.33	129.65	0.50	−2.77	127.83	1.61	−4.13
**7**	Liquiritin	33.33	33.10	1.04	−0.70	33.26	1.74	−0.21
		133.33	134.85	0.62	1.14	132.80	1.42	−0.40
		266.67	270.51	1.27	1.44	270.41	1.33	1.40
**8**	Isoliquiritin apioside	4.17	4.16	0.69	−0.11	4.20	1.34	0.76
		16.67	16.50	0.57	−0.97	16.56	0.74	−0.62
		33.33	33.33	0.99	−0.02	33.81	1.41	1.42
**9**	Benzoylmesaconine	2.08	2.09	0.43	0.20	2.11	1.14	1.35
		8.33	8.48	0.88	1.75	8.53	1.80	2.30
		16.67	16.45	0.74	−1.30	15.90	2.93	−4.58
**10**	Isoliquiritin	4.17	4.16	0.40	−0.08	4.15	0.93	−0.52
		16.67	16.77	1.03	0.59	16.63	0.96	−0.20
		33.33	33.46	1.05	0.39	34.36	2.28	3.07
**11**	Ononin	16.67	16.88	0.36	1.29	16.87	2.19	1.24
		66.67	69.58	0.89	4.37	68.93	1.89	3.40
		133.33	135.54	1.03	1.65	134.66	1.32	1.00
**12**	Benzoylaconine	0.52	0.53	1.29	1.30	0.53	1.06	1.30
		2.08	2.17	0.88	4.31	2.16	1.45	3.65
		4.17	4.14	0.61	−0.54	4.07	1.89	−2.30
**13**	Liquiritigenin	1.04	1.05	0.67	0.79	1.06	1.41	1.99
		4.17	4.23	0.58	1.53	4.35	2.26	4.45
		8.33	8.22	1.12	−1.41	8.09	1.71	−2.95
**14**	Echinatin	0.52	0.51	0.81	−1.54	0.52	1.28	−0.95
		2.08	2.06	1.30	−0.93	2.08	1.76	−0.10
		4.17	4.03	1.77	−3.38	4.09	2.25	−1.93
**15**	Genistein	0.52	0.52	1.43	−0.89	0.52	1.67	−0.90
		2.08	2.11	1.15	1.38	2.12	1.53	1.84
		4.17	4.03	0.90	−3.16	4.06	1.90	−2.54
**16**	Isoliquiritigenin	0.26	0.27	1.02	2.26	0.27	1.91	3.03
		1.04	1.06	1.60	1.28	1.08	2.96	3.96
		2.08	2.04	0.81	−2.17	2.02	2.66	−2.90
**17**	Formononetin	0.26	0.26	0.99	1.28	0.26	1.30	1.44
		1.04	1.06	0.94	1.98	1.07	1.45	2.87
		2.08	2.07	0.51	−0.75	2.02	2.04	−2.87
**18**	Glycyrrhizic acid	66.67	65.20	1.30	−2.19	65.97	1.72	−1.04
		266.67	257.30	2.54	−3.51	260.61	1.98	−2.27
		533.33	522.38	0.83	−2.05	526.07	1.42	−1.36
**19**	6-Gingerol	16.67	15.97	0.76	−4.21	16.25	1.67	−2.47
		66.67	66.26	0.74	−0.61	66.88	0.98	0.33
		133.33	126.84	1.69	−4.87	126.13	2.36	−5.41
**20**	Glabridin	1.04	1.02	1.26	−2.46	1.01	1.73	−3.26
		4.17	4.19	0.93	0.66	4.18	1.73	0.31
		8.33	8.07	0.91	−3.18	8.03	2.16	−3.65
**21**	8-Gingerol	1.04	1.04	0.51	−0.02	1.05	1.10	0.79
		4.17	4.20	0.92	0.77	4.30	2.39	3.17
		8.33	8.11	0.59	−2.72	7.96	2.14	−4.52
**22**	6-Shogaol	2.08	2.06	0.67	−0.96	2.10	1.99	0.87
		8.33	8.53	1.38	2.38	8.52	1.49	2.21
		16.67	16.10	1.53	−3.41	16.02	1.44	−3.88
**23**	Diacetoxy-6-gingerdiol	1.04	1.04	0.73	−0.19	1.05	1.35	0.35
		4.17	4.22	1.17	1.17	4.28	1.65	2.80
		8.33	8.02	1.50	−3.82	8.04	1.66	−3.47
**24**	8-Shogaol	0.52	0.52	0.91	−0.29	0.52	0.85	0.32
		2.08	2.11	0.47	1.30	2.12	1.03	1.71
		4.17	3.89	1.06	−6.52	4.01	2.85	−3.78

^a^ CV: coefficient of variation. ^b^ RE: relative error.

**Table 5:** Recovery data for the 24 compounds in SYT.

No.	Compound	Original Conc. (ng/mL)	Spiked Conc. (ng/mL)	Observed Conc. (ng/mL)	Recovery (%) ^a^	CV (%)
**1**	Karacoline	0.52	0.26	0.79	105.16	2.94
			1.04	1.67	110.24	2.48
			4.17	5.27	114.05	2.11
**2**	Fuziline	0.34	0.26	0.64	114.55	3.37
			1.04	1.50	111.37	2.43
			4.17	5.00	111.91	1.64
**3**	Bullatine B	0.71	0.52	1.29	110.77	2.69
			2.08	3.02	110.58	1.89
			8.33	10.19	113.67	1.55
**4**	Talatisamine	0.29	0.26	0.59	112.84	2.28
			1.04	1.49	114.57	2.88
			4.17	5.13	116.01	2.08
**5**	Liquiritin apioside	101.62	33.33	135.51	101.67	1.47
			133.33	257.50	116.91	2.28
			533.33	730.29	117.87	1.57
**6**	Neoliquiritin	16.43	8.33	25.81	112.54	2.73
			33.33	54.18	113.24	3.19
			133.33	173.47	117.78	1.58
**7**	Liquiritin	20.26	16.67	37.49	103.37	2.70
			66.67	95.60	113.00	1.43
			266.67	328.27	115.50	1.24
**8**	Isoliquiritin apioside	5.95	2.08	8.12	104.34	2.78
			8.33	15.55	115.26	1.98
			33.33	45.32	118.11	0.60
**9**	Benzoylmesaconine	1.53	1.04	2.75	117.07	2.87
			4.17	6.19	111.96	2.71
			16.67	20.52	113.94	1.43
**10**	Isoliquiritin	2.86	2.08	5.03	103.98	3.13
			8.33	12.52	115.96	1.74
			33.33	41.96	117.31	1.03
**11**	Ononin	10.92	8.33	20.63	116.47	1.68
			33.33	50.20	117.82	0.91
			133.33	169.68	119.07	0.43
**12**	Benzoylaconine	0.81	0.26	1.08	102.68	4.75
			1.04	1.92	106.26	2.38
			4.17	5.36	109.00	1.67
**13**	Liquiritigenin	1.02	0.52	1.57	105.70	2.73
			2.08	3.31	110.19	3.35
			8.33	10.53	114.15	1.59
**14**	Echinatin	0.11	0.26	0.39	108.10	3.70
			1.04	1.24	108.17	2.78
			4.17	4.61	108.14	2.58
**15**	Genistein	0.10	0.26	0.41	118.22	1.86
			1.04	1.25	110.53	2.35
			4.17	4.65	109.37	2.53
**16**	Isoliquiritigenin	0.12	0.13	0.26	107.60	1.42
			0.52	0.69	109.15	2.06
			2.08	2.46	112.06	1.47
**17**	Formononetin	0.08	0.13	0.24	117.86	3.19
			0.52	0.67	112.19	2.64
			2.08	2.35	108.77	0.63
**18**	Glycyrrhizic acid	92.53	33.33	123.99	94.39	4.13
			133.33	242.88	112.76	2.13
			533.33	682.41	110.60	0.82
**19**	6-Gingerol	14.48	8.33	22.84	100.39	4.27
			33.33	49.39	104.74	4.56
			133.33	168.54	115.55	2.26
**20**	Glabridin	0.56	0.52	1.06	96.84	3.81
			2.08	2.78	106.63	2.53
			8.33	9.97	112.97	2.71
**21**	8-Gingerol	0.96	0.52	1.47	96.63	2.62
			2.08	3.12	103.40	2.43
			8.33	9.96	107.99	2.51
**22**	6-Shogaol	1.69	1.04	2.68	94.88	1.33
			4.17	6.08	105.44	3.12
			16.67	20.26	111.43	1.47
**23**	Diacetoxy-6-gingerdiol	0.35	0.52	0.94	112.04	2.67
			2.08	2.64	109.82	2.43
			8.33	9.65	111.58	2.07
**24**	8-Shogaol	0.31	0.26	0.57	101.39	1.17
			1.04	1.48	112.77	1.28
			4.17	5.09	114.80	1.85

^a^ Recovery (%) = (Observed concentration − Original concentration)/Spiked concentration × 100.

**Table 6:** Contents of the 24 compounds in SYT extracts.

**No.**	**Compound**	**Batch 1**		**Batch 2**		**Batch 3**	
		**Mean ± SD** **(mg/g)**	**CV (%)**	**Mean ± SD** **(mg/g)**	**CV (%)**	**Mean ± SD** **(mg/g)**	**CV (%)**
**1**	Karacoline	0.027 ± 0.001	2.429	0.025 ± 0.001	2.842	0.025 ± 0.001	3.213
**2**	Fuziline	0.018 ± 0.000	2.179	0.018 ± 0.000	1.057	0.018 ± 0.000	1.356
**3**	Bullatine B	0.039 ± 0.001	2.080	0.037 ± 0.001	1.489	0.037 ± 0.001	3.156
**4**	Talatisamine	0.014 ± 0.000	1.936	0.013 ± 0.000	1.862	0.013 ± 0.000	1.852
**5**	Liquiritin apioside	6.882 ± 0.051	0.746	6.933 ± 0.064	0.923	6.870 ± 0.055	0.802
**6**	Neoliquiritin	0.885 ± 0.010	1.125	0.824 ± 0.017	2.063	0.814 ± 0.021	2.554
**7**	Liquiritin	1.303 ± 0.010	0.782	1.324 ± 0.023	1.709	1.331 ± 0.015	1.093
**8**	Isoliquiritin apioside	0.396 ± 0.005	1.237	0.399 ± 0.005	1.232	0.395 ± 0.004	1.125
**9**	Benzoylmesaconine	0.080 ± 0.001	1.555	0.077 ± 0.001	1.848	0.076 ± 0.002	2.829
**10**	Isoliquiritin	0.180 ± 0.002	1.322	0.182 ± 0.002	1.292	0.183 ± 0.002	1.081
**11**	Ononin	0.637 ± 0.013	2.030	0.599 ± 0.014	2.410	0.599 ± 0.010	1.628
**12**	Benzoylaconine	0.042 ± 0.000	1.133	0.038 ± 0.001	3.608	0.039 ± 0.001	3.746
**13**	Liquiritigenin	0.056 ± 0.001	1.695	0.054 ± 0.001	1.499	0.054 ± 0.001	1.941
**14**	Echinatin	0.006 ± 0.000	2.574	0.006 ± 0.000	2.992	0.006 ± 0.000	2.908
**15**	Genistein	0.008 ± 0.000	1.668	0.008 ± 0.000	1.894	0.008 ± 0.000	2.455
**16**	Isoliquiritigenin	0.006 ± 0.000	1.751	0.005 ± 0.000	3.293	0.005 ± 0.000	2.738
**17**	Formononetin	0.004 ± 0.000	1.441	0.004 ± 0.000	2.809	0.004 ± 0.000	2.144
**18**	Glycyrrhizic acid	5.540 ± 0.106	1.919	5.418 ± 0.157	2.891	5.505 ± 0.101	1.833
**19**	6-Gingerol	0.686 ± 0.006	0.929	0.649 ± 0.008	1.204	0.651 ± 0.013	2.073
**20**	Glabridin	0.031 ± 0.001	2.176	0.031 ± 0.001	2.103	0.031 ± 0.001	2.387
**21**	8-Gingerol	0.058 ± 0.001	1.848	0.056 ± 0.002	2.719	0.057 ± 0.001	1.376
**22**	6-Shogaol	0.097 ± 0.002	1.746	0.095 ± 0.001	1.391	0.095 ± 0.002	1.879
**23**	Diacetoxy-6-gingerdiol	0.020 ± 0.000	0.848	0.020 ± 0.000	0.472	0.020 ± 0.000	0.691
**24**	8-Shogaol	0.017 ± 0.000	2.243	0.017 ± 0.000	1.802	0.016 ± 0.000	1.834

## Data Availability

The data presented in this study are available in the article.
